# Spatial analysis of learning and developmental disorders in upper Cape Cod, Massachusetts using generalized additive models

**DOI:** 10.1186/1476-072X-9-7

**Published:** 2010-02-12

**Authors:** Kate Hoffman, Thomas F Webster, Janice M Weinberg, Ann Aschengrau, Patricia A Janulewicz, Roberta F White, Verónica M Vieira

**Affiliations:** 1 Department of Environmental Health, Boston University School of Public Health, Boston, USA; 2 Department of Biostatistics, Boston University School of Public Health, Boston, USA; 3 Department of Epidemiology, Boston University School of Public Health, Boston, USA

## Abstract

The spatial variability of three indicators of learning and developmental disability (LDD) was assessed for Cape Cod, Massachusetts. Maternal reports of receiving special education services, attention deficit hyperactivity disorder, and educational attainment were available for a birth cohort from 1969-1983. Using generalized additive models and residential history, maps of the odds of LDD were produced that also controlled for known risk factors. While results were not statistically significant, they suggest that children living in certain parts of Cape Cod were more likely to have a LDD. The spatial variation may be due to variation in the physical and social environment.

## Background

The prevalence of learning and developmental disabilities (LDDs) has increased over the last four decades [1,2]. Current prevalence estimates suggest that 5 to 15% of children in the United States are afflicted with disorders of learning and development, including disorders of memory, reduced IQ, attention deficit hyperactivity disorder (ADHD), autism spectrum disorders, conduct disorders and developmental delays [2-5]. The increased prevalence of LDDs has largely been attributed to improved diagnosis; however, evidence also suggests that the underlying prevalence of dysfunction is increasing [2]. Although the specific mechanisms for learning and developmental disorders remain unclear, much research has been conducted to identify their risk factors. These studies have consistently found that males and children from economic or culturally disadvantaged backgrounds have greater risk of being diagnosed with an LDD [1,6]. Numerous pre-birth risk factors have also been identified including lack of prenatal care; increased maternal age; pre-term delivery; low birth weight and prenatal alcohol, tobacco, and drug use [7-10]. Additional research suggests that the development of learning and developmental disorders is affected by intrinsic causes such as genetic differences in brain structure or biochemical imbalances [11]. However, it has also been suggested that the increased prevalence is attributable, at least in part, to chronic exposures to environmental toxicants [12-15].

Brain development begins in utero and continues throughout adolescence. This lengthy developmental period, coupled with its extreme complexity, leaves the developing brain particularly susceptible to adverse effects of chemical exposures [16]. Subtle changes in either structure or function can lead to profound neurological consequences that can persist over a lifetime. Prenatal and early childhood exposures to a number of environmental toxicants such as lead, methylmercury and polychlorinated biphenyls (PCBs) have been associated with damage to children's developing brains and nervous systems, and have been linked to specific neurological deficits and disorders [4,12,13,17-30]. While the neurotoxicological effects of a select few chemicals are fairly well characterized, relatively little is known about the 80,000 chemicals registered for commercial use with the Environmental Protection Agency [20].

The evolution of geographical information systems (GIS) and statistical methods has increased the use of spatial analyses to investigate the association between geographically-based exposures such as environmental contaminants and the risk of LDD. A number of studies have used residential proximity to a contaminant source to define exposure and assess the risk of neurological impairment associated with potential neurotoxicants [31-33]. Using this method, Dahlgren and colleagues found evidence that exposures to wood processing waste chemicals increase the severity of neurological symptoms reported in children and adults and was associated with impaired performance on neuropsychological tests [31].

While cluster analysis does not prove causation, it can serve as a useful tool in generating hypotheses about potential exposures that warrant further investigation [34]. In Binghamton, New York, Margai and Henry utilized two cluster detection methods, the Morans I Statistic and the Spatial Scan Statistic, to identify a spatial cluster of learning disability cases associated with historically significant sources of lead (i.e. automobile-related facilities and industries that dealt with lead and other byproducts) [35]. Conclusions were limited, however, because analyses were based on census block group prevalence and the investigators were unable to adjust for individual-level confounders.

New methods of spatial epidemiology involving generalized additive models (GAMs) permit analysis of point-based data while adjusting for individual-level variables [36,37]. The current research assesses the spatial variability of learning and developmental disorders in a cohort residing in the upper Cape Cod region of Massachusetts. Two general indicators of cognitive and behavioral function are addressed: receiving special education services for academic or behavioral difficulties and educational attainment. ADHD, a specific neurodevelopmental disorder, is also a focus of this study. Residential history data is used to examine potential differences in susceptibility during the prenatal and early childhood periods.

## Materials and methods

### Outcome Data

**Figure 1:**
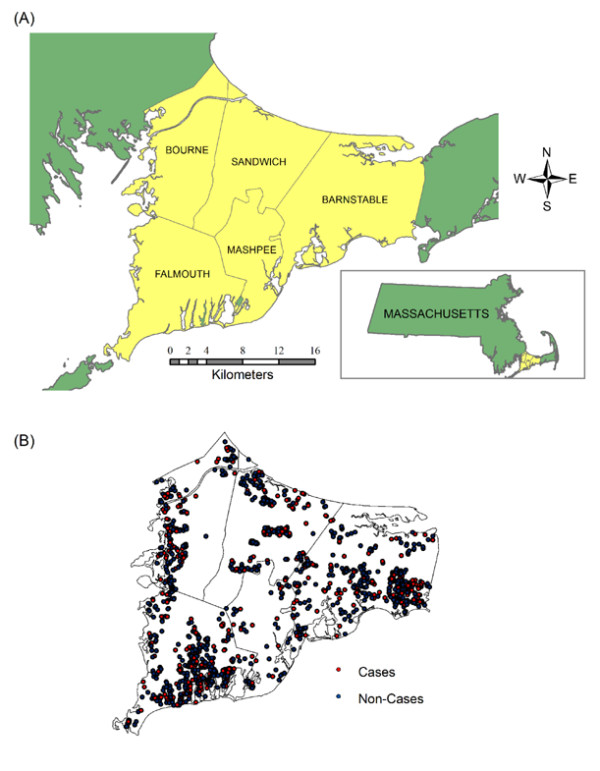
**Upper Cape Cod study area**. (A) Cape Cod is located in Massachusetts in the northeastern United States. (B) Distribution of participants' residences by outcome status. Locations have been altered to preserve confidentiality.

Data were collected as part of a large retrospective cohort study examining the association between exposure to tetrachloroethylene (PCE)-contaminated public drinking water and the risk of reproductive and developmental disorders in upper Cape Cod, MA [38]. Eligible families had at least one child born while they were living in the upper Cape Cod towns of Barnstable, Bourne, Falmouth, Mashpee, or Sandwich between 1969 and 1983 (Figure 1a). Birth certificates and questionnaires completed by mothers (or fathers, if mothers were unavailable) were used to gather information on outcome variables as well as potential confounders.

Children were excluded if they came from a multiple pregnancy; died before the age of 21 years; had a history of lead poisoning, fetal alcohol syndrome, mental retardation, cerebral palsy; or their mothers reported prenatal exposure to a known teratogen, daily or weekly marijuana use, or seven or more drinks of alcoholic beverages per week during the prenatal period. The final population was comprised of 1,574 subjects (Table 1; Figure 1b). To avoid clustering within families, analyses included only the eldest enrolled child in each family.

**Table 1:** Selected characteristics of case and non-case children.

Variable	Special Education		Educational Attainment		ADHD	
	**N = 1538**^**a**^		**N = 1542**^**b**^		**N = 1535**^**c**^	
	Cases	Non-Cases	Cases	Non-Cases	Cases	Non-Cases
	N (%)	N (%)	N (%)	N (%)	N (%)	N (%)
Male	186 (66.9)	592 (47.0)	219 (66.8)	562 (46.7)	105 (74.5)	673 (48.3)
Female	92 (33.1)	668 (53.0)	124 (36.2)	637 (53.1)	36 (25.5)	721 (51.7)
Birth Weight						
<2500 g	8 (2.9)	32 (2.5)	12 (3.5)	29 (2.5)	3 (2.1)	38 (2.7)
>= 2500 g	270 (97.1)	1228 (97.5)	331 (96.5)	1170 (97.5)	138 (97.9)	1356 (97.3)
Gestational Duration						
<37 Weeks	15 (5.4)	50 (4.0)	14 (4.1)	51 (4.3)	8 (5.7)	57 (4.1)
>= 37 Weeks	263 (94.6)	1210 (96.0)	329 (95.9)	1148 (95.7)	133 (94.3)	1332 (95.9)
Maternal Cigarette Smoking During Pregnancy						
Yes	81 (29.1)	331 (26.3)	116 (33.8)	296 (24.7)	41 (29.1)	370 (26.6)
No	194 (69.8)	910 (72.2)	220 (64.1)	887 (74.0)	99 (70.2)	1002 (71.9)
Missing	3 (1.1)	19 (1.5)	7 (2.1)	16 (1.3)	1 (0.7)	22 (1.6)
Maternal Alcoholic Beverage Consumption During Pregnancy						
Yes	99 (35.6)	487 (38.7)	111 (32.4)	475 (39.6)	55 (39.0)	527 (37.8)
No	175 (62.9)	750 (59.5)	224 (65.3)	703 (58.6)	85 (60.2)	839 (60.2)
Missing	4 (1.4)	23 (1.8)	8 (2.3)	21 (1.8)	1 (0.7)	22 (1.6)
Maternal Race						
White	270 (97.1)	1205 (95.6)	327 (95.3)	1152 (96.1)	136 (96.5)	1335 (95.8)
Other	8 (2.9)	55 (4.4)	16 (4.7)	47 (3.9)	5 (3.5)	59 (4.2)
Maternal Education						
<High School	13 (4.7)	39 (3.1)	31 (9.0)	23 (1.9)	4 (2.8)	48 (3.4)
High School	115 (41.4)	435 (34.5)	183 (53.3)	362 (30.2)	44 (31.2)	505 (36.2)
Some College	71 (25.5)	404 (32.0)	82 (24.0)	396 (33.0)	39 (27.7)	434 (31.1)
4 Year College Grad	79 (28.4)	382 (30.3)	47 (13.7)	418 (34.9)	54 (38.3)	407 (29.2)
Paternal Occupation						
Blue Collar	94 (33.8)	376 (29.8)	114 (33.2)	329 (27.4)	39 (27.7)	426 (30.6)
White Collar	118 (42.4)	643 (51.0)	143 (41.7)	651 (54.3)	71 (50.3)	690 (49.5)
Other	63 (22.7)	221 (17.5)	83 (24.2)	199 (16.6)	30 (21.3)	256 (18.4)
Missing	3 (1.1)	20 (1.6)	3 (0.9)	20 (1.7)	1 (0.7)	22 (1.6)

^a ^Missing special education information N = 36.

^b ^Missing educational attainment information N = 32.

^c ^Missing ADHD information N = 39.

Several measures of learning and developmental disabilities were collected by self-administered questionnaires, including maternal report of diagnoses of Attention Deficit Disorder (ADD) and Hyperactive Disorder (HD), difficulty sitting still or paying attention in school, difficulties or tutoring in reading and math, special education class placement for academic or developmental problems, repeating a grade, having an individualized education plan (IEP), receiving special education class placement, and educational attainment. Three binary outcome measures of learning and developmental disabilities, which were thought to be the least subject to misclassification of the outcome, are examined here.

### Special Education Placement

The first objective measure was based on maternal report of children receiving special education services for academic or behavioral difficulties. Since 1975, special education has been mandated under the Individuals with Disabilities Education Act (IDEA), which requires provisions for specifically designed instruction for individuals with disabilities. Although a number of different conditions are included as disabilities under IDEA, in general, the delivery of special educations services is an indicator of learning and developmental disabilities [39].

All students receiving special education services, even those with mild disabilities, are required by law to have an individualized education plan (IEP) which outlines specific educational goals and special provisions to aid in their education. Special education placement was defined by either maternal report of special classroom placement for academic or developmental difficulties or assignment of an IEP. In most instances, both events were reported by mothers.

### Maternal Report of ADHD

The second outcome measure combined maternal reporting of ADD and HD. Attention deficit hyperactivity disorder is the most common neurodevelopmental disorder of childhood, with an estimated prevalence between 7 and 16% of US children [4,40]. Children diagnosed with ADHD comprise a heterogeneous population sharing common symptoms, including inattention, impulsivity, and, in some cases, hyperactivity, or a combination of symptoms. As with other learning and developmental disorders, ADHD risk is influenced by biological, reproductive, environmental, and nutritional factors [41].

Until 1994, the Diagnostic Statistical Manual (DSM) listed ADD and HD as separate conditions [42]. Beginning with the DSM-IV, both outcomes were included as sub-types of the same condition, Attention Deficit Hyperactivity Disorder (ADHD) [43]. Research suggests that structural abnormalities in the frontal cortex and temporal lobe observed in both ADD and HD are similar [44]. For these reasons, reports from the questionnaire of ADD or HD were combined into a single outcome for the purposes of this study.

### Educational Attainment

The third outcome measure was highest level of educational attainment. Educational attainment is influenced by a number of factors related to learning and developmental disability, including low academic achievement, behavior problems, absenteeism, grade retention, and low parental educational expectations [45,46]. Although educational attainment is not a direct measure of learning or developmental disorder, the two outcomes are highly correlated [47,48]. Educational attainment data were divided into two categories: (1) completing high school or less education and (2) education beyond high school.

### Residential History Data

Mothers were asked to provide a residential history from before the birth of the child to age 18 years. All eligible addresses in the study area were geocoded using GIS and each residence was assigned corresponding latitude and longitude coordinates. The residential history was used to examine location at different developmental stages. To reflect exposures during the prenatal period, analyses were based on the residential location at birth. As early childhood chemical exposures are also thought to impact LDD risk, we conducted a second set of analyses including the residence from birth through age five years. Multiple residences for the same individual, however, may generate an artificial spatial cluster caused by a single case moving within a small area. To avoid potential bias due to the inclusion of multiple residences for the same individual, we used the address with the longest duration of residence from birth to age 5 years [36].

### Statistical Analyses

The log odds of each indicator of learning and developmental disability were estimated using generalized additive models, an extension of linear models that can incorporate both nonparametric and parametric model components and can be used to analyze binary outcome data [49]. For non-parametric model components, GAMs replace the beta coefficients of an ordinary logistic regression with a smooth term. In these analyses, a bivariate smooth was applied to latitude and longitude coordinates representing geographic location. Covariates were modeled as parametric terms.

A locally weighted regression smoother (loess), which adapts to differences in population density across the study area was used. The circular region or neighborhood from which data are drawn to predict the smooth is based on the percentage of data points in the neighborhood (with weighting based on distance from the center) and is referred to as the span size. Choice of span size produces a trade off between bias and variability. A large span size results in a smoother surface with low variability, but increased bias. Conversely, choosing a small span size results in high variability and comparatively low bias. To determine the optimal amount of smoothing in each analysis, Akaike's Information Criterion (AIC) was minimized [49].

A rectangular grid covering the study area was generated using the minimum and maximum latitude and longitude coordinates of study participant residences (approximate grid cell size 0.1 km^2^). Using GIS, a map of the study area was used to clip out points where participants could not live, such as water bodies or conservation land. At each grid point within the study area, an odds ratio (OR) was calculated using the entire study area as the referent group; the odds at each point was divided by the odds from a reduced model which omitted the latitude and longitude smooth term. Odds ratios were mapped using a continuous color scale (dark blue to dark red). A constant scale range was used for all maps of a particular outcome.

For each outcome, the null hypothesis that learning and developmental disability risk does not depend on location (e.g. the map surface is a horizontal plane) was tested. Locations of individuals were permuted 999 times while preserving their outcome status and covariates. For each permutation, the GAM with the optimal span size from the original dataset was run and the deviance statistic computed. If this global statistic indicated that location was statistically significant at p-value < 0.05, point-wise departures from the null hypothesis were evaluated using the distribution of the log odds at each point from the same set of permutations used to calculate the global statistic. Areas with significantly increased and decreased log odds were defined to include all observed points ranking in the upper and lower 2.5% of the point-wise distribution respectively.

A detailed discussion of the statistical methods of mapping population-based case control data using GAMs is provided by Webster and colleagues [37]. Statistical analyses were performed in S-Plus using the *gam *package and a local scoring algorithm GAM estimation procedure [49,50]. All maps were created using ArcGIS 9.2 [51].

Potential confounders were assessed based on the *a priori *expectation of their association with learning and developmental disorders. Drawing from existing research, sex, race, birth weight, gestational duration, year of birth, socioeconomic status (represented by mother's educational attainment and father's occupation), maternal alcohol or tobacco use during pregnancy, and PCE exposure were included as core confounders to include in all analyses. Spatial confounding was assessed by visually comparing maps with and without adjustment. The following variables did not change the appearance of the maps and were dropped from all presented models: mother's age, breastfeeding, maternal history of learning difficulties, occasional maternal marijuana use during pregnancy, pregnancy complications, prenatal exposure to solvents, and iron and multivitamin supplementation during pregnancy. Children with missing covariate data were excluded from the analyses (less than 2.1% for maternal smoking during pregnancy, 2.5% for maternal alcohol during pregnancy, and 1.7% for paternal occupation). Available characteristics of excluded children, including outcome prevalence, were very similar to children without missing data.

## Results

### Special Education Services

**Figure 2:**
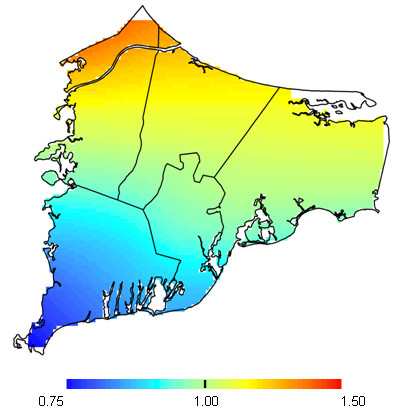
**Special education service results for birth address**. Fully adjusted model. The optimal span is 0.95. The map was not significantly different from flat (p = 0.57).

Of the 1,484 children with complete outcome and covariate data, 270 (18.2%) received special education services. In adjusted models, children receiving special education services were more likely to be male (OR = 2.24; 95% CI 1.71-2.96) and from lower SES families (highest maternal educational attainment compared to lowest OR = 0.79; 95% CI 0.54-1.15). For analyses using either the birth address or early childhood address the optimal span size was 0.95 (Table 2). Maps produced from both sets of analyses were similar. We show results of the adjusted birth address analysis in Figure 2. Each map showed an area of increased risk of needing special education services in the northern area of the town of Bourne but the difference was not statistically significant (birth address p-value = 0.57; early childhood p-value = 0.38). Maps with and without adjustment were similar indicating that spatial confounding was not an issue (data not shown).

**Table 2:** Summary of special education services models (270 Cases, 1214 Non-Cases).

Analysis	**Span **^**a**^	**Deviance p-value**^**b**^	Figure Number
Adjusted ^c^ Birth Residence	0.95	0.57	2

Crude Birth Residence	0.95	0.30	---

Adjusted ^c^ Early Childhood Residence	0.95	0.38	---

Crude Early Childhood Residence	0.95	0.22	---

^a ^Optimal span obtained by using the Akaike' Information Criterion (AIC).

^b ^Null hypothesis is that the map is flat.

^c ^Adjusted for sex, race, birth weight, gestational duration, year of birth, socioeconomic status, maternal alcohol or tobacco use during pregnancy, and PCE exposure.

### ADHD

**Figure 3:**
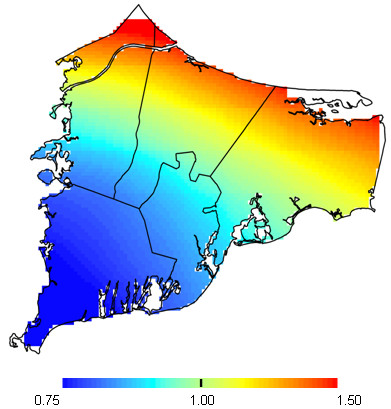
**ADHD results for birth address**. Fully adjusted model. The optimal span is 0.95. The map was not significantly different from flat (p = 0.33).

The reported prevalence of ADHD in the study population was 9.4% (139 cases; 1340 non-cases; Table 3), consistent with other United States population estimates during the same time period [40]. As maps of ADHD were similar using birth address and early childhood address, the former is shown (Figure 3). The risk of ADHD appears slightly elevated in the northern region of upper Cape Cod, but the difference was not statistically significant (optimal span size = 0.95; p-value = 0.33). Adjusting for potential spatial confounders made no appreciable difference in the map of ADHD risk, suggesting that spatial confounding was not an issue (data not shown).

**Table 3:** Summary of ADHD models (139 Cases, 1340 Non-Cases).

Analysis	**Span **^**a**^	**Deviance p-value**^**b**^	Figure Number
Adjusted ^c^ Birth Residence	0.95	0.33	3

Crude Birth Residence	0.95	0.17	---

Adjusted ^c^ Early Childhood Residence	0.95	0.35	---

Crude Early Childhood Residence	0.95	0.32	---

^a ^Optimal span obtained by using the Akaike' Information Criterion (AIC).

^b ^Null hypothesis is that the map is flat.

^c ^Adjusted for sex, race, birth weight, gestational duration, year of birth, socioeconomic status, maternal alcohol or tobacco use during pregnancy, and PCE exposure.

### Educational Attainment

**Figure 4:**
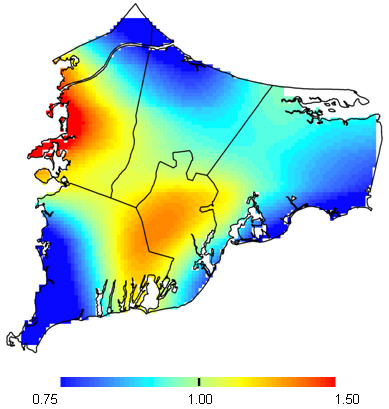
**Educational attainment results for early childhood addresses**. Fully adjusted model, optimal 0.60 span. The map was not significantly different from flat (p = 0.22).

Mothers reported that 22.3% of their children did not receive education beyond high school (Table 4). Boys were more likely to have low educational attainment (OR = 2.18; 95% CI 1.66-2.87) as were children from lower SES families (father employed in blue collar verse white collar occupations OR = 1.64; 95% CI 1.20-2.26). Using early childhood address, the adjusted analysis for the risk of low educational attainment appeared elevated in areas of Bourne and Mashpee (optimal span size = 0.60, p-value = 0.22; Table 4; Figure 4). The pattern of the risk surface was very similar using the birth address when the same span size was used for both analyses (not shown). Changes in the span size between the crude and adjusted models (optimal span sizes of 0.15 and 0.60 respectively) were primarily due to confounding by SES variables (mother's educational attainment).

**Table 4:** Summary of educational attainment models (331 Cases, 1155 Non-Cases).

Analysis	**Span **^**a**^	**Deviance p-value**^**b**^	Figure Number
Adjusted ^c^ Birth Residence	0.95	0.89	---

Crude Birth Residence	0.20	0.002	---

Adjusted ^c^ Early Childhood Residence	0.60	0.22	4

Crude Early Childhood Residence	0.15	<0.001	---

^a ^Optimal span obtained by using the Akaike' Information Criterion (AIC).

^b ^Null hypothesis is that the map is flat.

^c ^Adjusted for sex, race, birth weight, gestational duration, year of birth, socioeconomic status, maternal alcohol or tobacco use during pregnancy, and PCE exposure.

## Discussion

The risk of two indicators of learning and developmental disorders--special education placement and ADHD--was elevated in the Bourne and Falmouth areas of upper Cape Cod; however, the variability was not statistically significant (alpha = 0.05). The risk of low educational attainment was elevated in portions of Mashpee and Bourne, but not statistically significant. Spatial variability of the outcomes was evaluated using two periods of residence, reflecting prenatal residence and early childhood residences (before age 5 years). The similarity of the results--when evaluated using the same span size--was likely related to residential stability; less than one third of subjects moved during this period. Consequently, each analysis was based on many of the same addresses.

The spatial investigation approach used in this study has a number of strengths. Unlike previous spatial analyses of LDD, it was possible to use point-based data and avoid potential biases due to data aggregation within arbitrary political boundaries [35]. Additionally, it was possible to adjust for numerous covariates to assess potential spatial confounding. While it is often assumed that spatial confounding produces disease clusters, adjusting for covariates in these analyses made little difference in maps which we attribute to the relative homogeneity of the upper Cape Cod study population. Lastly, using residential history data enabled the examination of LDD risk during the prenatal and early childhood periods which are likely more relevant to the etiology of learning and development disorder risk than exposures associated with the address at diagnosis.

This investigation also has several limitations. Indicators of learning and developmental disability were obtained through maternal report. However, Faraone and colleagues found that maternal report of ADHD in particular was highly reliable (sensitivity and specificity, 95 and 97% respectively) [52]. In this cohort, 48 mothers (18% of cases) reported that their child was placed in special education but did not report that their child had an IEP. Because special education placement indicates more severe dysfunction, it is possible that mothers were more likely to remember special education class placement than the presence of an IEP. To reduce the misclassification of individuals with more severe dysfunction as non-cases by defining special education services by an IEP, all children whose mothers reported their child received any form of special education services were included as cases. Results were similar when we defined special education placement by the report of an IEP (data not shown).

We examined indicators of learning and developmental disability that were readily collected using a retrospective study design. However, these indicators are not sensitive enough to identify geographic areas where individuals experience more subtle dysfunction. ADHD, for example, is diagnosed using the number of symptoms and behaviors. A continuous outcome based on the number and severity of symptoms might have identified regions in upper Cape Cod where subclinical symptoms are elevated, even when a significant increase in ADHD diagnosis is not observed.

Residential history data allowed us to take into account location during different periods of development for individuals. Unfortunately, there were too few cases to perform more data-intensive space-time analyses as we have done previously with breast cancer in Cape Cod [53]. When possible, consideration of both location and time of exposure may generate hypotheses on short term environmental exposures.

The optimal tradeoff between bias and variance of the smooth (e.g. the optimal span size) was determined by minimizing the AIC. Selecting the optimal span for a given dataset, however, may obscure important map features at smaller scales. Examining different span sizes may reveal important features of the data. Similarly, p-values were used to evaluate global spatial variation as well as local areas of increased or decreased risk. Confidence intervals might be considered more informative, but the information is difficult to show graphically for surfaces. Permutation tests were conducted using the optimal span size of the observed data. Although the optimal span size was generally large for these data, permuted datasets may have had a larger optimal span size under the null hypothesis that the map is flat. The effect of using the optimal span of the observed data for permuted data could result in a p-value that is too small. This is a topic of future research.

Although results were not statistically significant, spatial variation was suggested in the study area. If real, there may be several possible explanations, including residual confounding at the individual level by unmeasured variables, as well as differences in the social and physical environment. Although the pattern of risk was similar for ADHD and special education placement, maps of the risk of low educational attainment displayed a different pattern. Educational attainment may be a less specific indicator of an LDD than the other outcomes considered, which were based on maternal report of an outcome that required evaluation by school or health care professionals. Differences in the pattern of risk between the outcomes may also be related to differences in educational resources or other school-related factors. There were no school-based data available to test this hypothesis. In upper Cape Cod, town boundaries approximate public school district boundaries. If variability was due solely to school district related factors we would not have expected odds estimates to vary within towns. There may be other group-level variables of importance. Statistical methods are needed for combined spatial and multilevel analyses; we are currently working on this problem. The suggestive spatial variation in LDDs could also be related to environmental exposure. In the northern part of the study area, several hazardous waste sites as well as a power plant were identified in previous health studies [54]. As data on environmental exposures from these or other sources in the area were not available, further assessment of exposure may be warranted.

## Conclusions

These exploratory analyses produced maps of the risk of three indicators of LDD--special education placement, ADHD, and low educational attainment--in upper Cape Cod using GAMs and GIS. Results suggest that children living in certain parts of upper Cape Cod may have been more likely to have an LDD. The addresses used (birth addresses or childhood addresses) did not affect the spatial distribution of observed odds. Adjustment for known risk factors had little effect. Observed variation, although not statistically significant, may be due to local differences in the social or physical environment.

## List of Abbreviations

(AIC): Akaike's Information Criterion; (ADD): attention deficit disorder; (ADHD): attention deficit hyperactivity disorder; (DSM): Diagnostic Statistical Manual; (GAMs): generalized additive models; (GIS): geographical information systems; (HD): hyperactive disorder; (IEP): individualized education plan; (LDD): learning and developmental disability; (loess): locally weighted regression smoother; (OR): odds ratio; (PCBs): polychlorinated biphenyls; (PCE): tetrachloroethylene.

## Competing interests

The authors declare that they have no competing interests.

## Authors' contributions

KH conducted spatial analyses and drafted the manuscript. TW collaborated on all analytic and editorial decisions. JW provided statistical support and consulted on analytic and editorial issues. AA provided the data and assisted in the epidemiologic analysis and editing. PJ and RW provided guidance relevant to the epidemiologic investigation of learning and development disabilities and edited the manuscript. VV collaborated on all analytic and editorial decisions and provided support for spatial analyses. All authors have read and approved the final manuscript.
